# Characterization of *Klebsiella pneumoniae* bacteriophages, KP1 and KP12, with deep learning-based structure prediction

**DOI:** 10.3389/fmicb.2022.990910

**Published:** 2023-01-24

**Authors:** Youngju Kim, Sang-Mok Lee, Linh Khanh Nong, Jaehyung Kim, Seung Bum Kim, Donghyuk Kim

**Affiliations:** ^1^Optipharm Inc., Cheongju-si, Chungcheongbuk-do, Republic of Korea; ^2^Department of Microbiology and Molecular Biology, College of Biological Science and Biotechnology, Chungnam National University, Daejeon, Republic of Korea; ^3^School of Energy and Chemical Engineering, Ulsan National Institute of Science and Technology (UNIST), Ulsan, Republic of Korea

**Keywords:** *Klebsiella pneumoniae*, bacteriophage, comparative genomic analysis, lysis-associated protein, AlphaFold, 3D structure prediction, protein structure alignment

## Abstract

Concerns over *Klebsiella pneumoniae* resistance to the last-line antibiotic treatment have prompted a reconsideration of bacteriophage therapy in public health. Biotechnological application of phages and their gene products as an alternative to antibiotics necessitates the understanding of their genomic context. This study sequenced, annotated, characterized, and compared two *Klebsiella* phages, KP1 and KP12. Physiological validations identified KP1 and KP12 as members of *Myoviridae* family. Both phages showed that their activities were stable in a wide range of pH and temperature. They exhibit a host specificity toward *K. pneumoniae* with a broad intraspecies host range. General features of genome size, coding density, percentage GC content, and phylogenetic analyses revealed that these bacteriophages are distantly related. Phage lytic proteins (endolysin, anti-/holin, spanin) identified by the local alignment against different databases, were subjected to further bioinformatic analyses including three-dimensional (3D) structure prediction by AlphaFold. AlphaFold models of phage lysis proteins were consistent with the published X-ray crystal structures, suggesting the presence of T4-like and P1/P2-like bacteriophage lysis proteins in KP1 and KP12, respectively. By providing the primary sequence information, this study contributes novel bacteriophages for research and development pipelines of phage therapy that ultimately, cater to the unmet clinical and industrial needs against *K. pneumoniae* pathogens.

## Introduction

*Klebsiella pneumoniae* is a Gram-negative opportunistic pathogen causing an extensive range of infections in the urinary tract, lungs, liver, abdominal cavity, and surgical sites in humans (Paczosa and Mecsas, 2016; Ssekatawa et al., 2021). In 2017, World Health Organization ranked this organism in the top three critical pathogens of international concern that must be prioritized for research, discovery, and development of new antibiotics (Govindaraj Vaithinathan and Vanitha, 2018). *Klebsiella pneumoniae* is of growing prevalence due to its resistance to a broad spectrum of drugs such as beta-lactam antibiotics, fluoroquinolones, aminoglycosides, and alarmingly, the last-line antibiotic treatment colistin (Dsouza et al., 2017; Manohar et al., 2019). The spread of multidrug-resistant *K. pneumoniae* and constant failure of antibiotic treatment have encouraged research for alternatives to antibiotics.

In both *in vitro* and *in vivo* models, bacteriophage therapy has reported promising outcomes as an alternative to reduce the reliance on antibiotics (Lin et al., 2017). Phage therapy indeed, has major advantages over antibiotics such as high diversity and abundance, host-specificity, work synergistically with other phages as a phage cocktail, low-cost preparations, and safe for human use (Furfaro et al., 2018). This practice utilizes lytic phages which selectively kill their respective bacterial hosts while leaving mammal cells intact hence, minimizing the risk of developing resistant commensal bacteria which is often an undesirable effect of antibiotic use. To date, there are several strategies for using phages against *K. pneumoniae*. Burn wound infections by *K. pneumoniae* in murine models had shown a significant improvement upon topical application of phage (Kumari et al., 2011), and administration of liposome-loaded phage cocktail (Chadha et al., 2017). Likewise, a study by Cao et al. has demonstrated a reduced lung inflammation and protection against lethal *K. pneumoniae* infection in mice by delivering intranasal phage (Cao et al., 2015).

Unlike strictly lytic phages, temperate phages are not the preferred candidates for phage therapy. Temperate phages can undergo a lysogenic life cycle by integrating their genome into the host and remain dormant as prophages. Prophages are either replicated along with the bacterial genome when cells divide or induced into a lytic life cycle upon exposure to environmental stress. Thus, if genome integration occurs, temperate phages as therapeutic agents might not give an instant bactericidal effect (Monteiro et al., 2019). There are also concerns about horizontal gene transfer that may leave the host with more virulence traits (Borodovich et al., 2022), and superinfection immunity in which the hosts become resistant to subsequent phage infections (Cumby et al., 2012). Despite these drawbacks, temperate phages hold several advantages, making them a great source of unexploited potential given the recent advances in bacteriophage engineering. Strategies such as temperate phage-antibiotic synergy (Al-Anany et al., 2021), temperate phage cocktail (Nale et al., 2018), engineering virulent mutant of temperate phage (Kilcher et al., 2018), and constructing a temperate phage-based delivery system to restore antimicrobial sensitivity (Edgar et al., 2012) have been evaluated. Regardless of the phage lifestyles, limitations of using whole phage entities have also been reported (Lin et al., 2022), giving rise to the considerations of phage-derived lytic proteins in therapy.

Several frontiers of using phage-derived lytic proteins with the ability to degrade major components of bacterial cell wall have been established (Oliveira et al., 2018; Wang et al., 2021). Endolysin, a class of enzymes that cleaves bacterial peptidoglycan (PG), has shown great potential as antimicrobials (Kong and Ryu, 2015). Due to the coevolution of the phage and host organism, it is reported that endolysins have also evolved to target the highly conserved structures of bacterial cell wall (Nelson et al., 2012; Young, 2014). Some endolysins exhibit near-species specificity which is an essential feature for their therapeutic application over classical antibiotics (Schmelcher et al., 2012). However, exogenous application of endolysin against Gram-negative bacteria has not been as successful as those on Gram-positive bacteria due to the differences in cell wall structures. The outer membrane (OM) in the Gram-negative bacteria forms a barrier that prevents endolysin from permeating to target the PG layer underneath. Thus, besides searching for native endolysins that can pass the OM by themselves, strategies such as combining OM permeabilizers treatment with endolysins, engineered lysins, and encapsulation into a carrier system have been proposed (Gontijo et al., 2021). To enable a rational design of endolysins with improved antibacterial efficacy, knowledge of their tertiary structures would be required.

The 3D protein structures have been mainly elucidated using X-ray diffraction of crystallized samples, cryo-electron microscopy, and single-particle analysis (Dubach and Guskov, 2020). However, a deep learning-based structure prediction algorithm termed AlphaFold which was recently released with its source code and model parameters (Jumper et al., 2021; Tunyasuvunakool et al., 2021), has facilitated protein structure prediction with remarkable accuracy using only the amino acid sequence of target protein.

Herein, this study reports a complete phenotypic and genomic characterization of two bacteriophages KP1 and KP12. Their potential for phage therapy were assessed based on the experimental confirmation of their physiological properties against *K. pneumoniae*, and the bioinformatics analyses including the models of their lytic proteins generated by AlphaFold with high confidence scores. Critical appraisal of novel bacteriophages not only would provide insights into the phage-host interactions but also facilitate research toward a non-toxic, non-invasive, and sustainable approach against bacterial resistance.

## Materials and methods

### Host bacteria and culture conditions

To isolate bacteriophages against *K. pneumoniae* producing extended-spectrum β-lactamase (ESBL(+) *K. pneumoniae*), # KPN10 strain (ID: K16-KPN-13-022) as the host bacteria was cultured in Tryptic Soy Broth (TSB, BD, Germany) at 35°C for 18 h. Other bacterial strains tested in this study were cultured in TSB at 35°C for 18 h.

### Bacteriophage isolation and purification

Because sewage is a reservoir of highly abundant enteric pathogens and novel bacteriophages (Alharbi and Ziadi, 2021; Gontijo et al., 2021), sewage samples were collected from a farm in Daejeon, South Korea. The samples were filtered through a 0.2 μm pore-size membrane filter (Sartorius, Germany). The filtrate (18 ml), 10X Tryptic Soy broth (TSB; 2 ml), and host bacteria culture (300 μl) were mixed and incubated at 35°C for 18 h. Following centrifugation at 4,000 rpm for 20 min, the supernatant was filtered through a 0.45 μm pore-size membrane filter. The filtrate (10 μl) was spotted on a Tryptic Soy Agar (TSA) plate with a lawn of *K. pneumoniae*. Plates were incubated at 35°C for 24 h and the lytic activity of bacteriophage was observed.

Soft agar overlay method (Hockett and Baltrus, 2017) was performed to purify the bacteriophage from the culture medium. The sample was diluted with SM buffer (100 mM NaCl, 10 mM MgSO_4_, 50 mM Tris–HCl [pH 7.5]) by 10-fold dilution. Diluted sample (100 μl), host bacteria culture (150 μl), and TSB top agar containing 0.6% agar (3 ml) were mixed and poured onto the TSA containing 2% agar plate followed by a 24 h incubation at 35°C. Single plaques were obtained, resuspended in 400 μl of SM buffer, and layered at room temperature for 4 h. The suspension (100 μl), TSB with 0.6% agar (12 ml), and host bacteria culture (300 μl) were mixed and layered on the TSA containing 2% agar bottom plate (diameter of 150 mm). Plates were incubated at 35°C for 24 h. To extract the bacteriophages, 15 ml of SM buffer was poured onto the cultured plate and stirred slowly at room temperature for 4 h. The suspension was recovered and centrifuged at 4,000 rpm, 4°C for 20 min. Then, the supernatant was filtered through a 0.22 μm pore-size membrane filter to obtain bacteriophage. To concentrate and purify lysates of the bacteriophage, polyethylene glycol 8,000 (PEG 8000, Sigma-Aldrich, United States) precipitation and purification *via* cesium chloride (CsCl, Sigma-Aldrich, United States) gradient ultracentrifugation were conducted. DNase 1 and RNase A (1 ug/ml) were added to phage lysate to degrade residual bacterial DNA and RNA during the reaction in 0.5 M NaCl at 40°C for 1 h. The mixture was centrifuged at 6,000 ×g, 4°C for 10 min. Phage-containing supernatant was precipitated by adding PEG 8000 to a final concentration of 10% w/v gradually with stirring at 4°C overnight. After centrifugation at 7,200 ×g, 4°C for 20 min, PEG-bacteriophage pellets were washed twice with 0.1 M ammonium acetate (pH 7.0), resuspended in 0.5 ml of sterile distilled water, and stored or incubated at 4°C overnight. Subsequently, bacteriophage suspension was purified using the CsCl gradient ultracentrifugation. The bacteriophage solution was injected in the CsCl gradient (*p* = 1.70, *p* = 1.50, *p* = 1.45, *p* = 1.30) followed by centrifugation at 78,500 ×g, 4°C for 2 h. The light-gray band was collected and dialyzed three times with membrane (SPECTRA/Pro 4 Dialysis membrane, United States) using 500 ml of SM buffer at 4°C. The purified bacteriophage suspension was stored at 4°C until further experiments.

To obtain the phage titers, soft agar overlay method with 10-fold serial dilutions of the purified phage lysates were performed and plaque forming unit (PFU) was calculated after incubation.

### Host specificity determination for bacteriophage

The host range of the isolated bacteriophages was tested against 11 ESBL(+) *K. pneumoniae* strains and nine other Gram-negative bacteria. The ESBL (+) *K. pneumoniae* strains were obtained from the Asian Bacterial Bank (ABB) of Asia Pacific Foundation for Infectious Diseases (APFID, Seoul, South Korea), which were all isolated from human blood. The other nine Gram-negative bacteria were *Acinetobacter baumannii* ATCC17978, *Citrobacter freundii* clinical isolate 15-0628, *Cronobacter sakazakii* KCTC 2949, *Escherichia coli* KCTC 1039, ESBL(+) *Escherichia coli* K01-ECO12-052, *Proteus mirabilis* KCTC2566, *Pseudomonas aeruginosa* KCTC2004, *Salmonella* Typhimurium ATCC14028, and *Salmonella* Enteritidis KCCM12021. All of these bacteria were handled by Optipharm Inc. using the standard soft agar overlay method for the host specificity test (Hockett and Baltrus, 2017). Briefly, fresh bacterial culture (150 μl) was inoculated with TSB 0.6% agar (3 ml), overlaid on TSA and spotted with 10 μl of the phage suspension. The plates were incubated at 35°C and observed for the presence of lysis plaques after 15 h. Lytic activity was determined based on the clarity of spots. EOP of phage was calculated as (average PFU on target bacteria/average PFU on host bacteria) × 100. The efficiency of plating (EOP) obtained on the host strain (K16-KPN-13-022) was considered as 100%. The experiment was conducted in triplicate.

### Electron microscopy imaging

Purified bacteriophage suspension (over 1.0 × 10^9^ PFU/ml) was applied to glow-discharged carbon-coated copper grids. After allowing the sample to absorb for 2 min, buffer solution was blotted off onto a Whatman paper and the sample on the grids were stained with 2% (w/v) uranyl acetate (UrAc) for 1 min. UrAc was blotted off. These results were recorded with the Tecnai G2 Spirit Twin microscope (FEI, United States) at an acceleration voltage of 120 kV.

### One-step growth curve analysis

The one-step growth curve analysis to determine the latent time and burst size was conducted as described previously (Pajunen et al., 2000), with some modifications. Briefly, 10 μl of bacteriophage suspension (approximately 1.0 × 10^9^ PFU/ml) was added to 10 ml of exponential phase culture of *K. pneumoniae* (1.0 × 10^8^ CFU/ml) to obtain the multiplicity of infection (MOI) of 0.1. The bacteriophage was allowed to adsorb onto the bacterial surface for 5 min at room temperature. Subsequently, the mixture was centrifuged at 10,000 ×g for 1 min and the resultant pellet was resuspended in 20 ml of TSB medium. Aliquots were collected at 5-min intervals, and the bacteriophage titer was measured with the standard double-layer top agar assay (Ellis and Delbruck, 1939).

### Analysis of bacteriophage proteins

Bacteriophage cultures were centrifuged with CsCl (Cesium Chloride, Sigma Aldrich, United States) gradient at 26,000 rpm for 4 h. Pellets collected were suspended with SM buffer to a concentration of 1.0 × 10^10^ PFU/ml. Then, 10 μl of 5X loading buffer was added to 40 μl of the phage suspension and boiled for 10 min. The mixture was loaded on a 12.5% SDS-PAGE gel and electrophoresis was performed (Boulanger, 2009).

### Bactericidal effect of bacteriophage

Approximately 10^8^ CFU/ml of *K. pneumoniae* culture was transferred to sterile tubes and infected with bacteriophages at different levels of MOI which were 10, 1, and 0.1. The tubes were incubated at 35°C with shaking for 3 h. Negative control (NC) was incubated without bacteriophage. The cell density (OD_600nm_) was measured at 1-h intervals using a spectrophotometer (Infinite M200PRO, TECAN, Swiss).

### Temperature and pH stability of bacteriophage

To determine the temperature stability of phages, KP1 and KP12 at a final concentration of 10^8^ PFU/mL were incubated at 40°C, 50°C, 60°C, and 70°C for an hour, phage titers were measured using the soft agar overlay method. To determine the phage stability at a range of pH, we used acetic acid and sodium acetate buffer for pH 2–6, phosphate buffer for pH 7–11, and Tris–HCl buffer for pH 8–11. The pH of buffers was adjusted with 1 M HCl or 1 M NaOH. Phage suspension (10 μl, 1.0 × 10^9^ PFU/ml) was added to each pH buffer (990 μl), gently mixed, and incubated at room temperature for 2 h. After treatment, phage titers were measured using the soft agar overlay method.

### Whole genome sequencing of bacteriophage

Bacteriophage suspension was treated with DNase I (TAKARA, Japan) to eliminate the host DNA. EDTA (20 mM), proteinase K (50 μg/ml, Qiagen), and SDS (0.5% w/v) were added to the bacteriophage suspension and allowed to stand at 50°C for an hour. The suspension was mixed with an equal volume of phenol-chloroform-isoamyl alcohol (25:24:1) and centrifuged at room temperature for 10 min. The recovered supernatant was again mixed with an equal volume of phenol-chloroform-isoamyl alcohol (25:24:1) and centrifuged at room temperature for 10 min. Subsequently, the supernatant was mixed with 10% (v/v) of the total volume of 3 M sodium acetate. A double volume of cold 95% ethyl alcohol was added followed by incubation at −20°C for an hour. Centrifugation was performed at 13,000 rpm for 10 min at 0°C. The resultant DNA pellet was washed twice with cold 70% ethyl alcohol (Deveau et al., 2002). After complete removal of ethyl alcohol, the pellet was dried and dissolved in 100 μl TE buffer (Tris-EDTA, pH 8.0). Whole genome sequencing of KP1 and KP12 was performed using the Illumina Hiseq2000 and PacBio RSII platform (Macrogen, Korea), respectively.

### Genome sequence analysis of KP1 and KP12

Genome sequences of both KP1 and KP12 were annotated using the Rapid Annotation Subsystem Technology toolkit (RASTtk) pipeline (Brettin et al., 2015). BLASTp analysis (90% coverage and 95% identity) were performed to assign putative functions for each predicted protein, based on its sequence homology against the National Center for Biotechnology Information (NCBI) non-redundant protein sequences database (NCBI-nr). Circular visualization of gene distribution was constructed using Circos program v0.69.6 (Krzywinski et al., 2009).

Subsequent analysis of phage-encoded lytic proteins (endolysin, holin, anti-holin, and i-/o-spanin) was conducted. To identify putative endolysin sequences, protein-encoding genes (PEGs) of KP1 and KP12 were queried against two different curated endolysin databases which were by Fernandez-Ruiz et al. (2018) and PHROGs (Terzian et al., 2021) using BLASTp with 85% minimum coverage, 25% minimum identity, and 1.00E-09 minimum BLAST E-value. To identify other lytic proteins, BLASTp was conducted against NCBI-nr and PHROGs. Multiple sequence alignment (MSA) of endolysin sequences were performed for KP1, KP12, and other 68 *Klebsiella* endolysin sequences annotated by RefSeq (MUSCLE alignment with default parameter and UPGMA clustering algorithm with 100 iterations).

### Phylogenetic analysis

As of June 2022, a total of 133 Reference Sequences (RefSeq) of bacteriophages infecting *Klebsiella* sp. have been deposited in the NCBI Virus database. These sequences include 5 *Ackermannviridae*, 45 *Autographiviridae*, 2 *Demerecviridae*, 33 *Drexlerviridae*, 31 *Myoviridae*, 3 *Podoviridae*, 4 *Schitoviridae*, and 10 *Siphoviridae.* Phylogenetic relationship of 135 bacteriophages including KP1 and KP12 at the amino acid level were evaluated using BPGA pipeline (USEARCH clustering tool with identity cut-off = 50%, MUSCLE alignment, and UPGMA algorithm; Chaudhari et al., 2016). A core genome-based phylogenetic tree generated by BPGA software was visualized *via* interactive Tree of Life (iTOL) v6 (Letunic and Bork, 2016).

### Pan-genome analysis

Pan-genomic studies determine the core (conserved), accessory (dispensable), and unique (strain-specific) gene pool of a species to uncover species evolution and relationship. Pan-genome analysis was performed concurrently with phylogenetic analysis using BPGA algorithm.

### Ortholog analysis between KP1 and KP12

Pairwise comparison between KP1 and KP12 was performed using PATRIC Proteome Comparison tool (Wattam et al., 2017)^1^ with default parameters (30% minimum coverage, 10% minimum identity, and 1.00E-05 minimum BLAST E-value) to identify the possible presence of orthologs between these strains.

### Protein structure prediction

Using the amino acid sequence of putative lytic proteins of KP1 and KP12, their structures were predicted using AlphaFold v2.0. AlphaFold v2.0 was locally installed together with multiple genetic databases which were UniRef90, MGnify, BFD, Uniclust30, PDB70, and PDB with mmCIF format. Structure prediction consisted of five steps which include MSA construction, template search, inference with five models, model ranking based on the average of per-residue confidence metric called predicted local distance difference test (pLDDT) score, and constrained relaxation of the predicted structures. For each target protein sequence, five structural models were generated. Each model was provided with its corresponding pLDDT score, predicted template modeling (pTM) score, and predicted aligned error (PAE) heatmap for the evaluation of model accuracy.^2^ Structural similarity of the predicted models to the experimental protein structures was calculated using TM-align—an algorithm for sequence-independent protein structure comparisons using heuristic dynamic programming based residue-to-residue alignment (Zhang and Skolnick, 2005).

### Expression and purification of putative endolysins

Gene encoding putative endolysin in KP1 (KP1.peg.110) was amplified by polymerase chain reaction (PCR) using primers KP1_F (5′-CATATGTTGAAACTTACGCTGGAACAA-3′) and KP1_R (5′-CTCGAGTTATTTTCCTTGATAAGCG-3′). Gene encoding putative endolysin in KP12 (KP12.peg.319) was amplified using primers KP12_F (5′-CATATGTTGAAACTTACGCTGGAACAA-3′) and KP12_R (5′-CTCGAGTTAAGAGGTTAGAACAGAT-3′). PCR was performed under the following conditions: one cycle of 10 min at 95°C, followed by 35 cycles of 90 s at 95°C, 90 s at an annealing temperature between 50°C and 66°C, 90 s at 72°C, and one final cycle of 10 min incubation at 72°C. The PCR products were cloned into the pGEM-T Easy Vector System (Promega, Madison, WI, United States), and *E. coli* transformants were selected on an LB agar plate containing ampicillin (50 μg/ml). Fragments encoding putative endolysins were recovered from the cloning vector using *Nde*I and *Xho*I [New England Biolabs (NEB), lpswich, MA, United States], and recloned into PET-28a expression vector (Novagen, Madison, WI, United States) to be expressed in *Escherichia coli* (*E. coli*) under 0.5-mM isopropyl β-D-1-thiogalactopyranoside (IPTG) induction. After cell disruption using a sonicator (Branson Advanced-Digital Sonifier 450 (20 kHz, 400 W), Branson Ultrasonics Corporation, Brookfield, CT, United States), putative endolysins were purified using Ni-NTA Agarose (Qiagen, Germany). Purified proteins were analyzed using 12% sodium dodecyl sulfate-polyacrylamide gel electrophoresis (SDS-PAGE) gels with a Precision Plus Dual Xtra Standard protein marker (Bio-Rad, Hercules, CA, United States).

### Spot test of the putative endolysins

Spot test was performed as described previously (Donovan et al., 2006). The overnight culture of *K. pneumoniae* (K16-KPN-13-022) was collected, washed once, and resuspended in PBS. The suspension was then autoclaved and centrifuged. The resulting pellet was resuspended in 200 μl of PBS and used as the substrate. The PBS solution contained 0.5 mM EDTA to increase the permeability of Gram-negative bacterial outer membrane according to the previous reference (Briers et al., 2014). The solution was added to 3 ml of TSB (0.6% agar) and overlaid on a TSA agar plate (2.0% agar). 10 μl of purified proteins was spotted on the overlaid plate and incubated at 37°C for 5 h. For negative control, samples using *E. coli* BL21(DE3) and *E. coli* DH5α were prepared *via* the same process.

## Results

### Experimental characterization of bacteriophage KP1 and KP12

**Figure 1:**
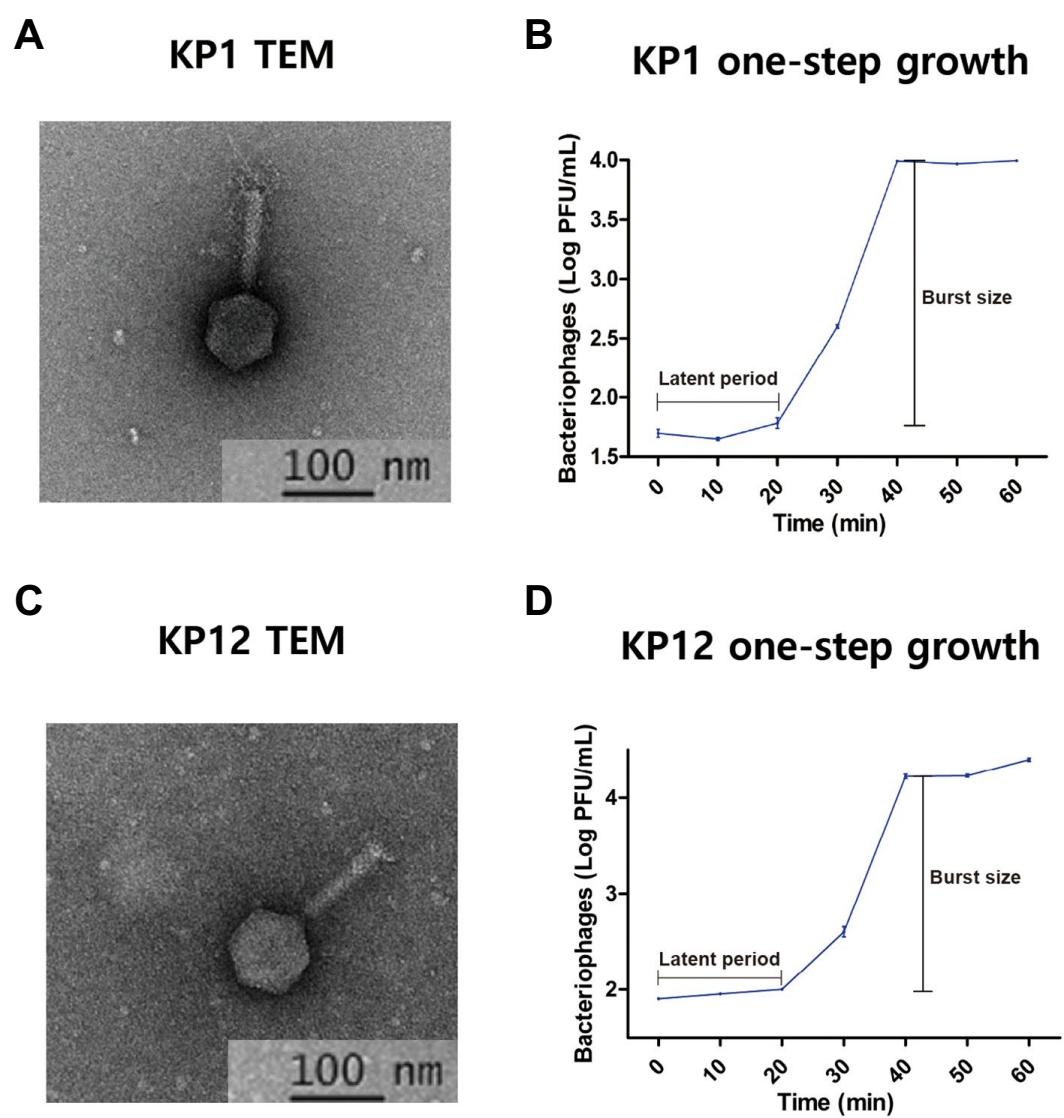
Isolated bacteriophages infecting *Klebsiella pneumoniae* KP1 and KP12. Transmission electron microphotographs (TEM) and one-step growth curves of KP1 **(A,B)** and KP12 **(C,D)**. The scale bars in each TEM represent 100 nm. TEMs indicate both phages show the classic form of *Myoviridae* with contractile tails (approximately 11 nm in diameter and 90 nm in length) and icosahedral heads (approximately 60 nm). The one-step growth curves for KP1 **(B)** and KP12 **(D)** show their latent periods estimated to be 20 min. The burst sizes of KP1 and KP12 were observed to be 197 and 312 PFU per infected cell, respectively.

Morphological analysis of KP1 and KP12 isolated from sewage samples revealed a classic form of *Myoviridae* bacteriophages with contractile tails and icosahedral heads. Each phage has a head diameter of approximately 60 nm and a tail with a diameter of 11 nm and length of 90 nm (Figures 1A, C). In the plaque assay, it was observed that both phages formed small plaques of about 1–2 mm diameter (Supplementary Figures S1A, C). One-step growth curves of KP1 and KP12 were constructed to determine their latent periods and burst sizes (Figures 1B, D). For each phage, a typical triphasic curve with lag phase, burst phase, and stationary phase was observed. The latent time of each phage was similar (about 20 min). The burst size of KP1 and KP12 was 197 and 312 PFU per infected cell, respectively. Structural proteins of phages were analyzed using SDS-PAGE (Supplementary Figures S1B, D). The molecular weights of these major proteins were compared with the size of PEGs in their genome annotation to postulate their functions. KP1 showed a pattern of six major protein bands including 70 kDa (terminase large subunit), 55 kDa (DNA ligase), 50 kDa (outer capsid protein), 35 kDa (head-tail protein), 33 kDa (thymidylate synthase), and 16 kDa (capsid and scaffold protein). The SDS-PAGE result of KP12 showed eight major proteins which are 74 kDa (DNA primase/helicase), 58 kDa (terminase large subunit), 43 kDa (phage major capsid protein), 25 kDa (putative baseplate assembly protein), 24 kDa (uncharacterized protein), 17 kDa (structural protein), 12 kDa (putative membrane protein), and 11 kDa (uncharacterized protein).

Subsequently, the host specificities of KP1 and KP12 were determined by their infectivity against various Gram-negative bacteria. Both KP1 and KP12 phages are highly specific for *K. pneumoniae* (Table 1). Out of 11 ESBL(+) *K. pneumoniae* strains tested, nine and eight bacterial strains were susceptible to KP1 and KP12, respectively. Lytic activity of phage against K01-KPN-13-149 and K20-KPN-12-057 strain was detected only in KP1 while K16-KPN-13-008 strain is only susceptible to KP12. K22-KPN-13-007 is resistant to both KP1 and KP12. The results indicated that KP1 and KP12 showed broad activity against ESBL(+) *K. pneumoniae*.

**Table 1:** Host specificity analysis of bacteriophage KP1 and KP12.

**Species**	**Strain**	**Phage KP1**		**Phage KP12**	
		**Spot testing**	**EOP (%)**	**Spot testing**	**EOP (%)**
ESBL(+) *Klebsiella pneumoniae*	K16-KPN-13-022	+	100	+	100
	K01-KPN-13-134	+	40.44 ± 1.25	+	49.08 ± 1.25
	K01-KPN-13-149	+	21.98 ± 2.62	−	0
	K07-KPN-13-002	+	97.65 ± 1.63	+	81.06 ± 3.40
	K14-KPN-13-016	+	83.39 ± 2.05	+	75.15 ± 2.16
	K16-KPN-13-008	−	0	+	51.73 ± 2.62
	K20-KPN-12-057	+	14.77 ± 2.49	−	0
	K20-KPN-12-067	+	44.30 ± 2.45	+	53.77 ± 2.45
	K21-KPN-12-013	+	62.42 ± 1.63	+	96.95 ± 0.94
	K22-KPN-13-007	−	0	−	0
	K22-KPN-13-013	+	14.60 ± 0.82	+	26.68 ± 1.70
**Other gram-negative bacteria**					
*Acinetobacter baumannii*	ATCC17978	−	0	−	0
*Citrobacter freundii*	Clinical isolate 15–0628	−	0	−	0
*Cronobacter sakazakii*	KCTC 2949	−	0	−	0
*Escherichia coli*	KCTC 1039	−	0	−	0
ESBL(+) *Escherichia coli*	K01-ECO12-052	−	0	−	0
*Proteus mirabilis*	KCTC2566	−	0	−	0
*Pseudomonas aeruginosa*	KCTC2004	−	0	−	0
*Salmonella* Typhimurium	ATCC14028	−	0	−	0
*Salmonella* Enteritidis	KCCM12021	−	0	−	0

+ with lytic activity, − without lytic activity. Efficiency of Plating (EOP) with the host strain K16-KPN-13-022 was considered as 100%.

**Figure 2:**
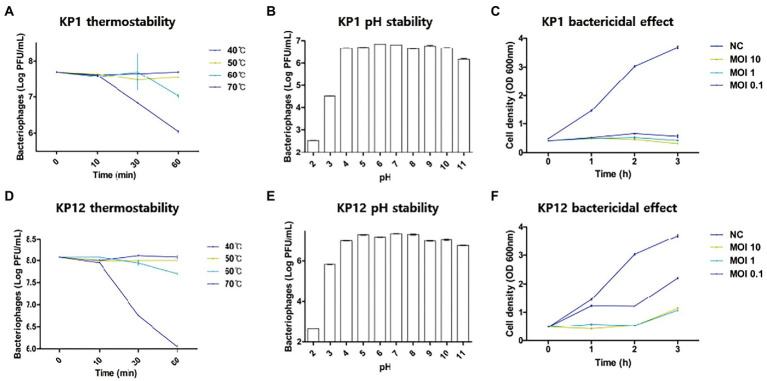
Physiological properties of KP1 and KP12. Effects of temperature, pH on the phage titer, and the phage bactericidal effects at different MOI levels of KP1 **(A–C)** and KP12 **(D–F)**. Thermostability of phages was tested in the range of 40°C–70°C for 60 min, and pH stability of phages was tested in the range of pH 2–11. To validate the bactericidal effect of each phage, bacteria were infected by KP1 and KP12 bacteriophage with MOIs of 10, 1, 0.1. Negative control (NC) was a bacteria-only culture media. Bacterial cell density on the phage titer with different MOI level was measured at 1-h intervals.

Since both bacteriophages showed infectivity against drug-resistant *K. pneumoniae*, thermostability and pH sensitivity of these phages were further investigated. Overall, the increase in temperature resulted in the decrease of bacteriophage titers (Figure 2) with the strongest reduction observed at 70°C. When compared to the starting phage titer (KP1: 7.70 ± 0.01 log_10_ PFU/ml, KP12: 8.07 ± 0.00 log_10_ PFU/ml), an over 100-fold reduction was observed in both phage titer after 60 min (KP1: 6.04 ± 0.03 log_10_ PFU/ml, KP12: 6.01 ± 0.01 log_10_ PFU/ml). At 60°C, both KP1 (7.02 ± 0.04 log_10_ PFU/ml) and KP12 (7.70 ± 0.01 log_10_ PFU/ml) showed titer reductions but less than those at 70°C. At 40°C and 50°C, phage titers remained relatively stable even after 1 h treatment. Regarding pH stability, the PFU counts (PFU/ml) of phage were over 1.0 × 10^6^ for both KP1 and KP12 which proved to be stable in the range of pH 4–11. At pH 3, there was a decline in both phage titers. However, the decrease observed in viable KP1 (Figure 2B) was lower as compared to KP12 (Figure 2E). At pH 2, KP1 and KP12 showed a notable reduction of approximately 2 log_10_ units and 3 log_10_ units in the phage titer, respectively. The lytic properties of KP1 (Figure 2C) and KP12 (Figure 2F) at different concentrations were investigated by analyzing the bacterial growth pattern for 3 h after phage infection. The negative control group (bacteria-only culture without phage) continued to grow during the incubation, finally reaching OD_600nm_ of about 4.0. In contrast, bacterial growth was inhibited upon phage infection. Specifically, the growth of *K. pneumoniae* was significantly inhibited when treated with KP1 phage regardless of the MOI levels. Upon infection with KP12 phage, the bacterial growth was inhibited for the first 2 h but recovered after that. Therefore, KP1 showed better infectivity against *K. pneumoniae* in which it efficiently inhibited the bacterial growth even at the lowest MOI level of 0.1.

### Genomic reconstruction of bacteriophage KP1 and KP12

**Figure 3:**
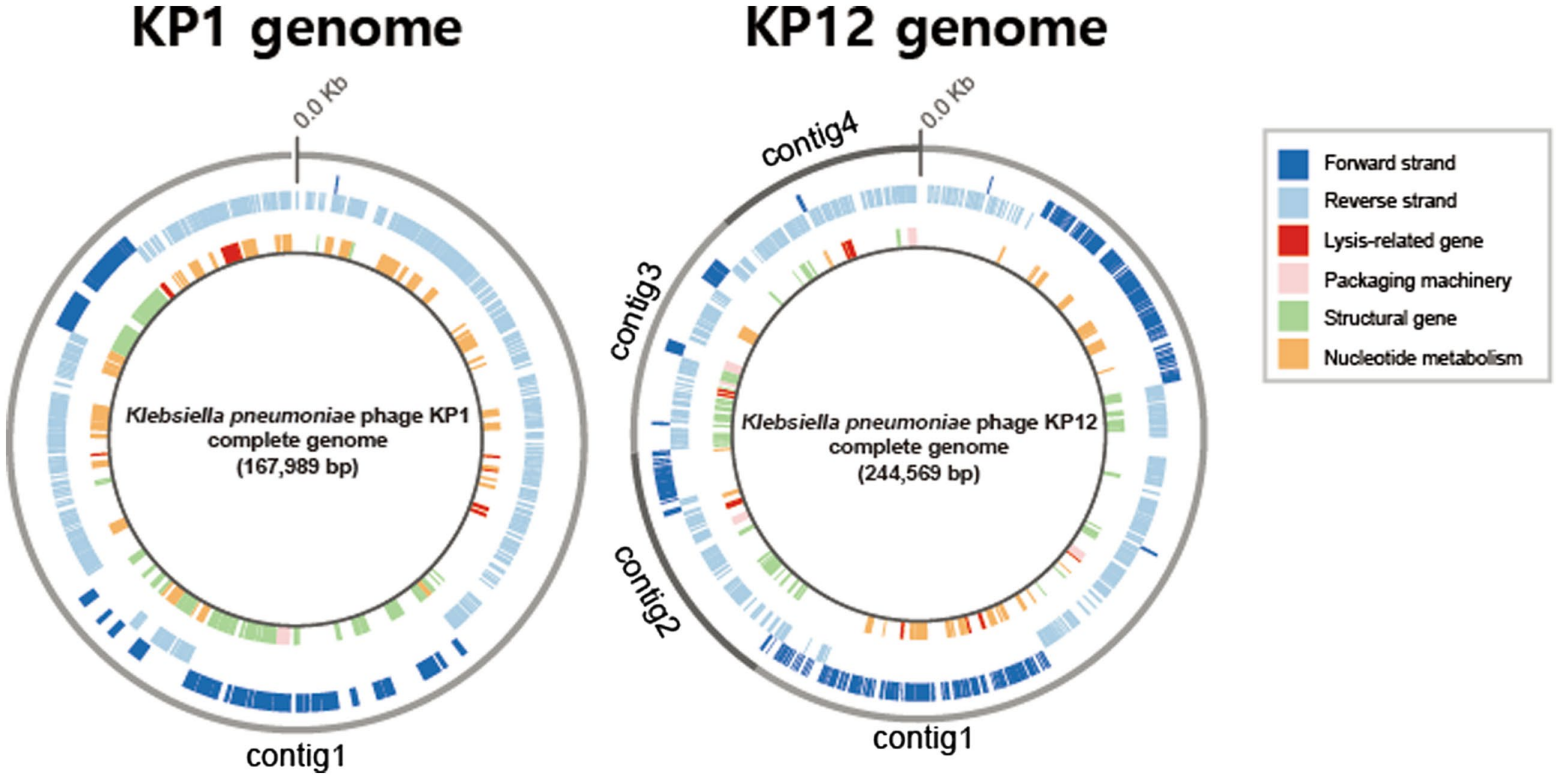
Circular representations of the bacteriophage KP1 and KP12 genomes. Outermost circle (gray) indicates the organization of contigs in this representation, followed by circles of forward (dark blue) and reverse (light blue) strands. Innermost circle are genes of putative functional categories, defined based on RASTtk annotation and are color coded (top right).

Basic genomic features and genes of both bacteriophages were annotated using the RASTtk pipeline. Based on its sequence homology, BLASTp analysis against the NCBI-nr database assigned putative functions for each predicted gene of KP1 and KP12. The 167,989 bp genome of KP1 is a linear contig with a coding density of 95.6%, and a GC content of 39.6% which is considerably lower than the content of its host *K. pneumoniae* (57.5%). The analysis of KP1 genome showed a total of 295 predicted genes. Of which, 281/295 were identified as PEGs and the remaining 14 genes were identified as tRNA coding genes (Supplementary Table S1). The genome sequence of KP12 was assembled into four contigs with a total length of 244,569 bp (Figure 3). With the genome size more than 200 kb, KP12 can be considered a jumbo phage (Yuan and Gao, 2017). Its coding density is 92.7% and GC content is 48.1%. Among the 414 putative genes of KP12, 397/414 and 17/414 genes were predicted to be PEGs and tRNA genes, respectively (Supplementary Table S1).

Apart from genes whose functions involved in phage packaging machinery, phage structures, and DNA replication, functional prediction revealed the presence of lytic-associated genes in both KP1 and KP12 (Figure 3). In addition to RASTtk and NCBI databases, phage lytic sequences (endolysin, anti-holin/holin, i-/o-spanin) were searched against the PHROGs database of prokaryotic virus proteins, allowing a more comprehensive update. The combined search approach predicted putative endolysin sequences in KP1 such as KP1.peg.110 and KP1.peg.142 whereas KP1.peg.242, KP1.peg.213, and KP1.peg.212 were identified as putative holin, i-spanin, and o-spanin, respectively (Supplementary Table S2). For KP12, sequences such as KP12.peg.198, KP12.peg.279, and KP12.peg.319 showed high sequence identity with endolysin while KP12.peg.280, KP12.peg.135 and KP12.peg.134 were identified as putative holin, i-spanin and o-spanin, respectively (Supplementary Table S3). Nonetheless, 44.5% of PEGs in KP1 (125/281) and 55.9% of PEGs in KP12 (222/397) have their functions remain unknown as they were being classified as hypothetical proteins, uncharacterized proteins, or domain of unknown function (DUF), highlighting the novelty of these bacteriophages.

### Phylogenetic and pan-genome analysis of *Klebsiella* bacteriophages

**Figure 4:**
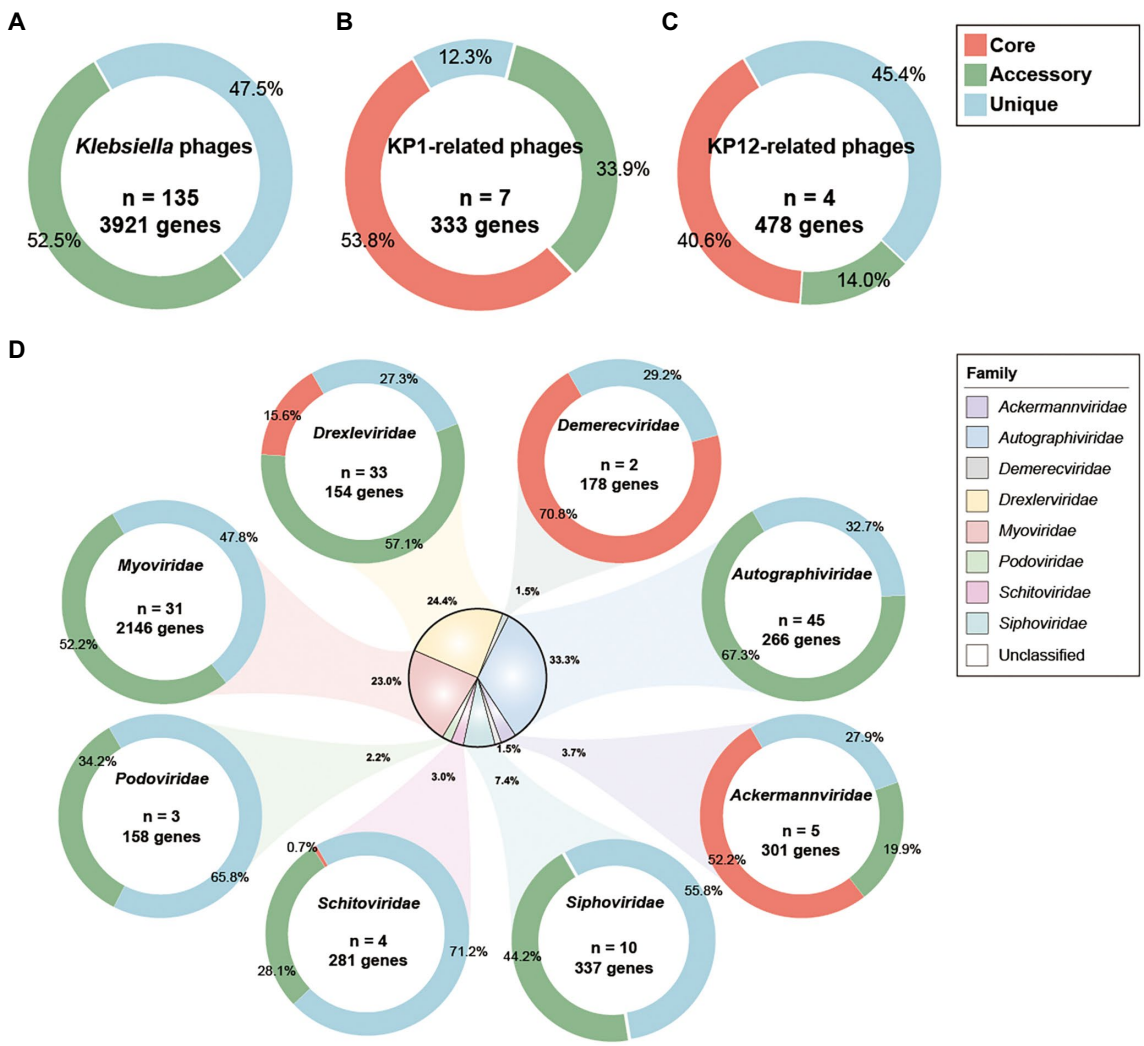
Pan-genome analysis of *Klebsiella* phages. Pan-genome analyses were performed for **(A)** 135 *Klebsiella* phages including KP1 and KP12, **(B)** KP1 and its closely related phages of *Myoviridae* family, **(C)** KP12 and its closely related phages of *Myoviridae* family, and **(D)** subgroup of 135 *Klebsiella* phages by their taxonomic family. The percentages of core (red), accessory (green), and unique genes (blue) were presented for each family. *n* indicates the number of strains for each family.

Pan-genomic analysis was introduced to investigate the characteristics of genomic features of KP1 and KP12 when compared with other bacteriophages infecting *K. pneumoniae*. Phylogenetic analysis showed bacteriophage KP1 and KP12 being clustered with members of the *Myoviridae* family (Supplementary Figure S2) agree with their morphological characteristics observed in TEM. However, KP1 and KP12 belong to distantly related clusters. To investigate the genetic similarity of KP1 and KP12 with other available *Klebsiella* phages, the core genome was determined from the constructed pan-genome of 135 strains in the dataset. No core genes were identified across all members of the examined population (Figure 4A). The non-linear regression analysis revealed an open pan-genome of *Klebsiella* phages (Supplementary Figure S3A). Likewise, the open pan-genome of 45 *Autographiviridae*, 10 *Siphoviridae*, three *Podoviridae*, and 31 *Myoviridae* showed no conserved genes (Figure 4D; Supplementary Figure S3). These results indicated that *Klebsiella* phages can constantly obtain foreign genes for adapting to different environments.

Pan-genomic analysis of KP1 and its closely related strains (JD18, Mineola, KPV15, vB KpnM KpV477, KP179, and PKO111) defined 179 core genes, 113 accessory genes, and 41 unique genes which account for 53.8%, 33.9%, and 12.3% of the pan-genome, respectively (Figure 4B). These strains share mostly genes responsible for DNA replication and structural proteins. Notably, they also share genes involved in host lysis such as phospholipase and tail lysozyme (Supplementary Table S4). Pan-genomic analysis of KP12 and its closely related strains (KpS8, BIS47, and KB57) showed 194 core genes, 67 accessory genes, and 217 unique genes which account for 40.6%, 14.0%, and 45.4% of the pan-genome, respectively (Figure 4C). Their representative core sequences mainly consist of hypothetical proteins, structural proteins, and ribonucleotide reductase. BPGA software also detected the presence of endolysin sequences in their core genome (Supplementary Table S5).

Conservation analysis was conducted to identify orthologous genes between KP1 and KP12 bacteriophages. The result indicated that they share only 14 orthologs, which accounted for approximately 5.0% of PEGs in KP1 (14/281) and 3.5% of PEGs in KP12 (14/397; Supplementary Table S6). Their representative orthologous genes mainly consist of hypothetical proteins, endonuclease, DNA/RNA ligase, structural proteins, and ribonucleotide reductase. This provides further evidence that the genetic backgrounds of KP1 and KP12 are different, supporting the result of phylogenetic analysis.

Sequence alignment of *Klebsiella* phage-derived endolysins revealed a vast diversity exists even among bacteriophages that target the same host. The highly conserved endolysins of KP1 (KP1.peg.110) and Mineola (YP_010096097.1) formed a lineage with a distinct sequence organization as compared to other phage clusters, while endolysin sequence of KP12 (KP12.peg.319) and of vB_KpnM_KBV79 phage are closely related (Supplementary Figure S4).

### Putative host lytic proteins of bacteriophage KP1 and KP12 based on the 3D protein structure modeling

Besides bacteriophages, the use of purified phage-derived lytic proteins is of particular interest to phage-based pharmaceuticals. Structure prediction of putative phage lysis proteins in KP1 and KP12 was performed using AlphaFold. In the subsequent TM-align analysis, the AlphaFold models generated were structurally aligned with the X-ray crystals of endolysin and anti-/holin to provide a more detailed comparison.

**Figure 5:**
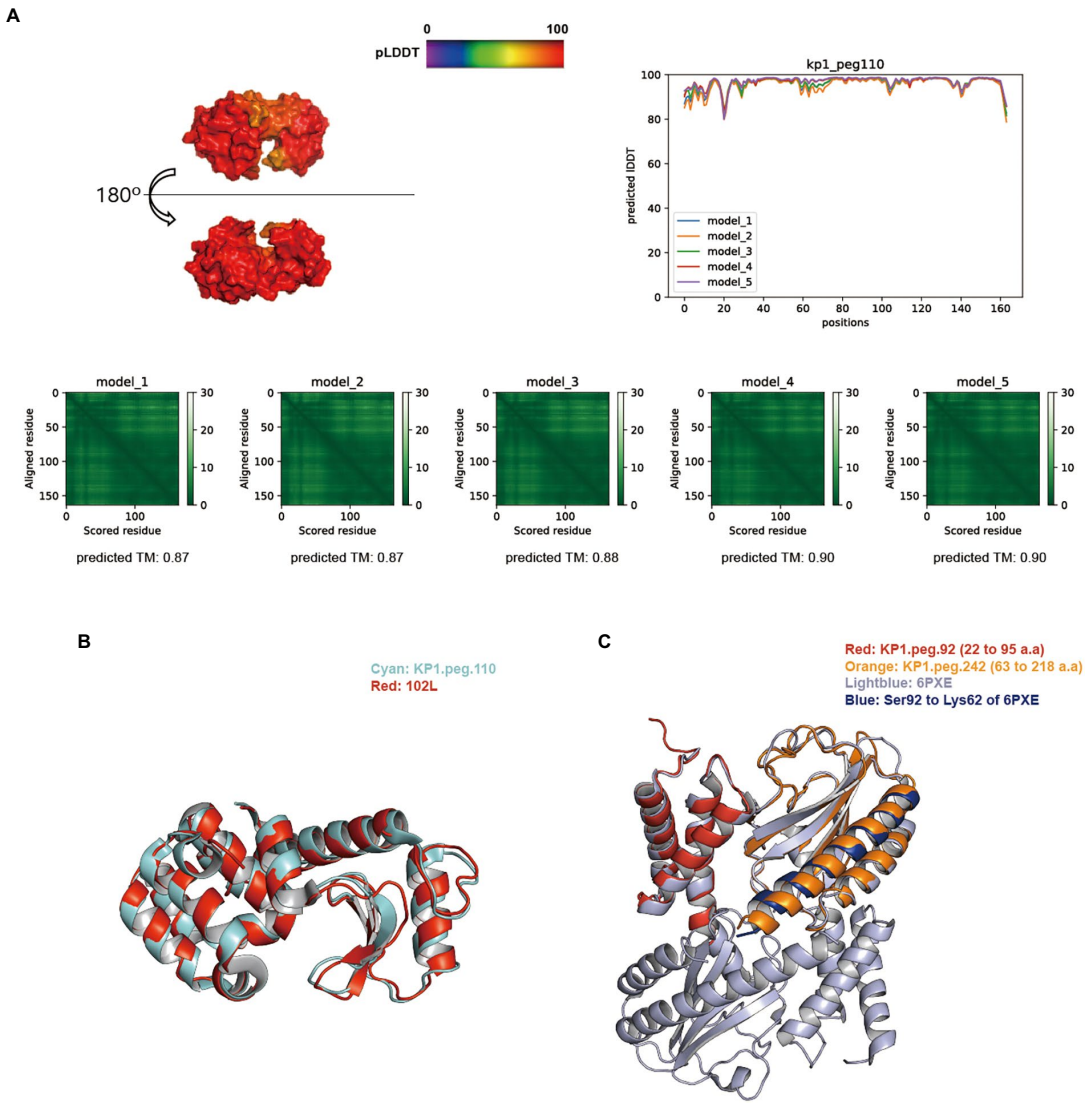
Structure prediction of putative host-lytic proteins in KP1 and their alignments with X-ray crystal structures. **(A)** AlphaFold models generated for KP1.peg.110 show high pLDDT and predicted TM-scores. Protein surface structure of the first pTM model (model_1; top left), and its corresponding 2D plot showing the score for amino acid at each position (top right) are color-coded according to its pLDDT. PAE heatmaps of five models and their pTM scores illustrate the predicted error between all pairs of residues. The lower value (darker green) indicates higher confidence in the prediction. **(B)** Structural alignment of the KP1.peg.110 model (cyan) to T4 endolysin E X-ray crystal structure (PDB ID: 102 l, red). **(C)** Structural alignment of the partial KP1.peg.92 (putative anti-holin, residue 22–95, red) and the partial KP1.peg.242 (putative holin, residue 63–218, orange) to T4 RI-T complex crystal structure (PDB ID: 6PXE, light blue). The continuous helix from Ser92 to Lys62 of 6PXE is colored blue.

BLASTp search against the NCBI, PHROGs, and curated endolysin databases identified KP1.peg.110, KP1.peg.142, KP12.peg.198, KP12.peg.279, and KP12.peg.319 as putative endolysin sequences. The overall distribution of pLDDT per residue and its average score of five models generated for KP1 putative endolysins were above 91, indicating models of high accuracy. In particular, the predictive models of KP1.peg.110 (Figure 5A) and KP1.peg.142 (Supplementary Figure S5A) had the pLDDT average scores of 96.23 ± 0.64 and 91.15 ± 1.43, respectively. The average of five predicted TM-scores of KP1.peg.110 (0.88 ± 0.01) and KP1.peg.142 (0.82 ± 0.03) were above 0.8. PAE heatmaps indicating the certainty of relative domain position in the predicted models showed confident single domain in both KP1.peg.110 and KP1.peg.142 models with KP1.peg.110 having relatively lower predicted errors.

**Figure 6:**
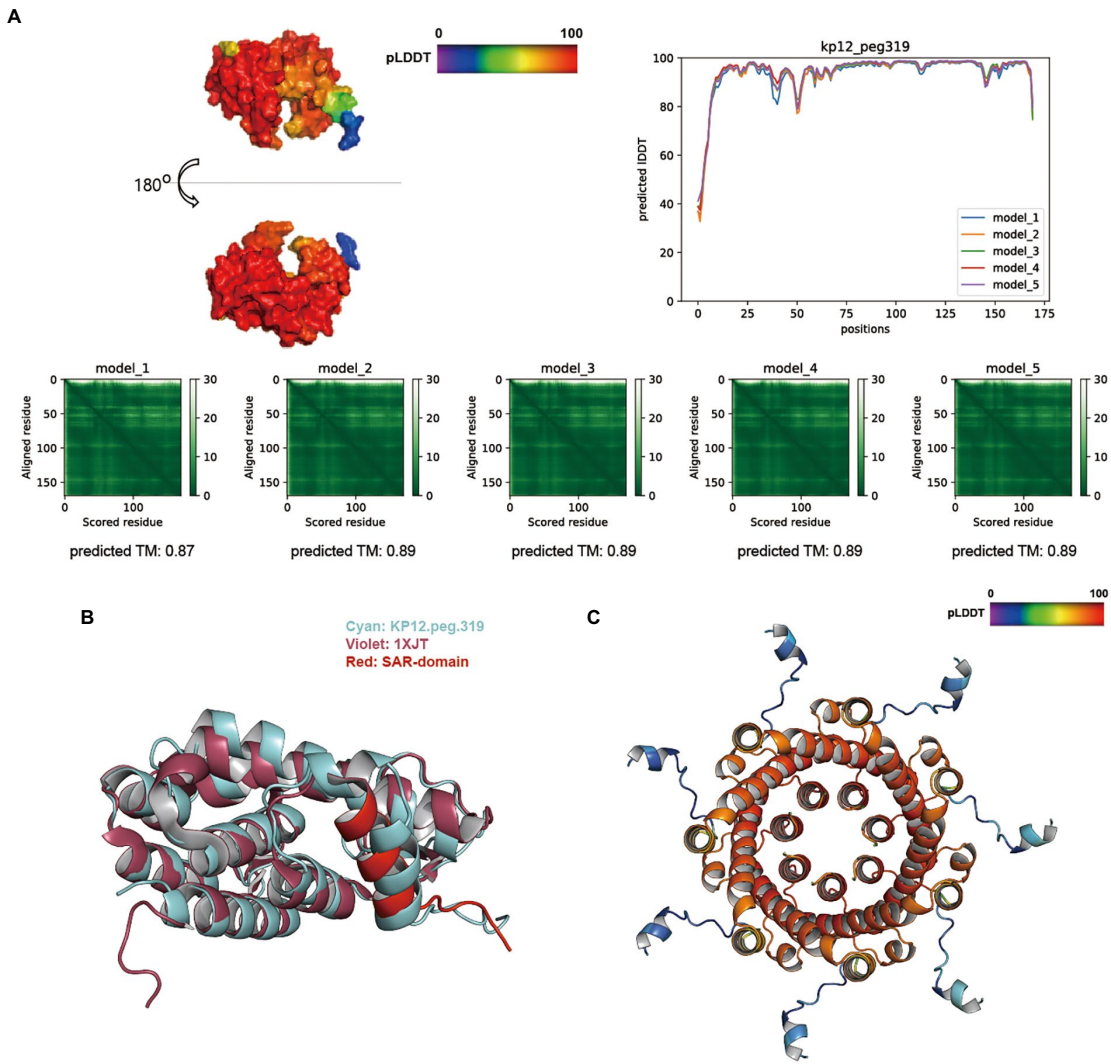
Structure prediction of putative host-lytic proteins in KP12 and their alignments with X-ray crystal structures. **(A)** AlphaFold models generated for KP12.peg.319 show high pLDDT and predicted TM-scores. Protein surface structure of the first pTM model (model_1; top left), and its corresponding 2D plot showing the score for amino acid at each position (top right) are color-coded according to its pLDDT. PAE heatmaps of five models and their pTM scores illustrate the predicted error between all pairs of residues. The lower value (darker green) indicates higher confidence in the prediction. **(B)** Structural alignment of the KP12.peg.319 model (cyan) to P1 endolysin Lyz (PDB ID: 1XJT, violet). The SAR domain of Lyz is marked in red. **(C)** The predictive homoheptamer of KP12.peg.280 (putative holin) is color-coded according to the pLDDT score of its pTM model_1.

The overall score distribution of pLDDT mean values was above 92 for KP12 putative endolysin sequences. Specifically, KP12.peg.319 (Figure 6A), KP12.peg.198 (Supplementary Figure S5B), and KP12.peg.279 (Supplementary Figure S5C) had their respective scores of 93.86 ± 0.57, 96.07 ± 1.28, and 92.83 ± 0.76. The average predicted TM-score of KP12.peg.319 (0.89 ± 0.01) and KP12.peg.198 (0.90 ± 0.01) were similar while the score of KP12.peg.279 was comparatively lower (0.77 ± 0.01). PAE heatmaps indicated that the models for all three sequences have confident individual domains. While KP12.peg.319 and KP12.peg.198 are most likely to have single domain, PAE plots of KP12.peg.279 exhibited two low-error regions corresponding to two domains where pLDDT score distribution was below 80 at the N-terminus (residue 1–50) and above 90 at the C–terminus (residue 60–240).

TM-align analysis was performed to structurally align and compare the predicted models to the biochemically verified coliphage endolysins (Supplementary Table S7). KP1.peg.110 showed a good structural similarity with the bacteriophage T4 endolysin (PDB ID: 102 l), with a TM-align score of 0.9630 (Figure 5B). In contrast, KP1.peg.142 matched poorly with 102 l (TM-align score: 0.2787) yet showed a high similarity with the phage T4 cell–puncturing device (PDB ID: 1 K28) with a TM-align score of 0.98305. Furthermore, homotrimer structure prediction of KP1.peg.142 identified a triple β-helix domain—a structure often found in the C-terminus of phage T4 baseplate protein Gp5 (Kanamaru et al., 2002; Weigele et al., 2003; Chao et al., 2022; Supplementary Figure S6A). Therefore, it is likely that KP1.peg.110 encodes for an endolysin whereas KP1.peg.142 encodes for a tail lysozyme (Supplementary Table S2). When predicting protein structure, the putative holin and anti-holin sequences were trimmed to match the removed region of X–ray crystallography images for protein overproduction and crystallization purposes (Krieger et al., 2020; Mehner-Breitfeld et al., 2021). Partial holin sequence (KP1.peg.242, residue 22–95) and partial anti-holin sequence of KP1 (KP1.peg.92, residue 63–218) matched well with the crystal structure of T4 bacteriophage RI (anti-holin)–T (holin) complex (PDB ID: 6PXE), with the respective TM-align scores of 0.84035 and 0.95489 (Figure 5C). The continuous helix domain of KP1.peg.242 showed a consistent structural alignment with 6PXE from Ser92 to Lys62.

Despite being predicted as putative endolysins, KP12.peg.198 and KP12.peg.279 returned relatively low TM-align scores (below 0.6) when aligned with coliphage endolysins (Supplementary Table S7). Instead, KP12.peg.198 showed a better structural similarity with other GH19 family endolysin (PDB ID: 4OK7) encoded in *Myoviridae* bacteriophage (*Salmonella* phage SPN1S), with a TM-align score of 0.70975. Nevertheless, KP12.peg.319 structure was highly similar to the *Myoviridae* P1 phage endolysin Lyz with a TM-align score of 0.83277 (Supplementary Table S7). Moreover, it was found that the helical signal-anchor for type II membrane protein and the single-arrest-release (SAR) domain of Lyz SAR endolysins (Xu et al., 2005) were conserved in KP12.peg.319 (Figure 6B). This prompted an investigation into the presence of its lytic partner pinholin in KP12. The homoheptamer of pinholin was previously reported to form nano-scale pinholes (Pang et al., 2009). In KP12, homoheptamer structure prediction of its putative holin sequences revealed that the pLDDT score distribution of KP12.peg.280 (73.15 ± 7.65) was higher than that of KP12.peg.320 (50.78 ± 6.08). Most importantly, the nano-scale channel formation was identified in the KP12.peg.280 predicted structure (Figure 6C).

Indeed, the typical lysis cassettes of T1–like phages and P1/P2–like phages were both found in the genome of KP12 (KP12.peg.278–280, KP12.peg.316–320). T1–like lysis cassette of KP12 (KP12.peg.278–280) showed the representative arrangement in the order of holin, endolysin, and spanin. However, the putative endolysin (KP12.peg.279) was predicted to have two domains which is not consistent with the small single-domain globular structure of Gram-negative bacteria endolysin. Moreover, its overall pLDDT score distribution at the N–terminus (residue 1–60) was low hence, the predicted structure was not reliable (Supplementary Figure S5C). In addition, unlike phage T1, the putative spanin of KP12 (KP12.peg.278) lacks the recognizable periplasmic β-sheet domain of unimolecular spanin (u-spanin). Instead, its 3D model (Supplementary Figure S6B) showed coiled-coil α-helices structure of i-spanin (Kongari et al., 2018). KP12.peg.316–320 showed a genetic architecture of *Myoviridae* P2 lysis cassette including the overlapped form of i-spanin (KP12.peg.317) and o-spanin (KP12.peg.316; Kongari et al., 2018). Since the X-ray crystallography of P2 bacteriophage endolysin K was unavailable, TM-align analysis against P2 endolysin was not performed for further verification. However, given that the endolysin sequence of KP12 (KP12.peg.319) showed a high TM-align score against the P1 endolysin Lyz, it can be inferred that KP12 is likely to be P1/P2-like bacteriophage.

To verify the bacteriolytic capacity of putative endolysins in KP1 (KP1.peg.110) and KP12 (KP12.peg.319), the genes were cloned and expressed using *E. coli* expression system with IPTG induction, followed by Ni-NTA based purification. SDS-PAGE showed that both purified proteins were of expected molecular weight (Supplementary Figure S7A, KP1.peg.110: 18.0 kDa; Supplementary Figure S7B, KP12.peg.319: 18.6 kDa). The purified proteins were then used for spot tests against ESBL(+) *Klebsiella pneumoniae* (K16-KPN-13-022), which demonstrated their lytic properties toward *K. pneumoniae* (Supplementary Figure S7C) but not for *E. coli* strains BL21(DE3) and DH5α (data not shown).

## Discussion

As an attempt to combat multidrug-resistant *K. pneumoniae*, the historical practice of bacteriophage therapy emerges as a promising alternative to the traditional antibiotics, due to their remarkable host specificity, hence minimizing the perturbance of microbiota in patients. In the present work, *Klebsiella* phages KP1 and KP12 were characterized and assigned as members of two distantly related *Myoviridae* clusters based on their TEMs and phylogenetic analyses. The lifestyle of the bacteriophages were additionally investigated by searching the presence of lysogeny-associated genes such as integrase and using several published prediction tools (e.g., PHACTS; McNair et al., 2012), PhagePred (Song, 2020), BACHPHLIP (Hockenberry and Wilke, 2021), and PhaTYP (Shang et al., 2022; Supplementary Table S8). In case of KP1, there was no temperate phage marker genes found in the BLASTp analysis (Supplementary Table S2), and there was a consistent prediction of KP1 as a lytic phage across the tools. For KP12, BLASTp search showed the presence of integrase (KP12.peg.344) and transposase (KP12.peg.288) in its genome (Supplementary Table S3) while the lifestyle prediction showed mixed results among tools. Considering the subsequent analysis of the KP12 lysis cassette and structural prediction of its host lysis proteins, KP12 could be considered a P1/P2-like temperate phage.

KP1 exhibits a close relation with *Klebsiella* phages JD18, KP179, KPV15, Mineola, PKO111, and vB_KpnM_KpV477 which formed a cluster that belongs to *Jiaodavirus* genus (*Tevenvirinae* subfamily, *Myoviridae* family; Komisarova et al., 2017), suggesting the grouping of KP1 to the sublineage shared by these phages. Since *Jiaodavrius* phages encode a Hoc-like protein which is highly immunogenic (Townsend et al., 2021), it would be insightful to investigate the immunogenicity of KP1. KP12 and its phylogenetically related phages including KpS8, vB_KpnM_BIS47, and vB_KpnM_KB57 were classified as genus *Mydovirus* of subfamily *Vequintavirinae*, thereby suggesting the classification of KP12 to this genus.

Pan-genome analysis revealing the core, accessory, and unique genes of *Klebsiella* phages may facilitate our understanding of their host-range specificity. Genetic diversity can be inferred from the number of genes that compound the core genome. A smaller core genome reflects the increasing diversity among the studied organisms. Computing the pan-genome of all available *Klebsiella* phages and within their respective families resulted in the open pan-genome with few conserved genes, indicating an extensive genomic diversity regardless of their common morphological features. The conserved genome was detected in two groups namely KP1 and phages of *Jiaodavirus* genus, KP12 and phages of *Mydovirus* genus. Their core genomes primarily constitute genes encoding essential structural and regulatory functions. Orthologous genes between KP1 and KP12 were also mainly genes encoding regulatory functions such as ligase or endonuclease.

Host range largely defines the utility of phage in therapy. The desirable phage should have a narrower host range in terms of bacterial species and a broader host range in terms of strains within the target species. For phage therapy, this would presumably result in fewer mismatched host-phage combinations, hence rendering a better treatment (Hyman, 2019). Although the current host range tests did not cover all 79 capsular types of *K. pneumoniae* (Pan et al., 2015), both phages showed high specificity against *K. pneumoniae* including ESBL (+) *K. pneumoniae* strains isolated from clinical environment with high morbidity and mortality rates associated (da Silva et al., 2021), indicating their potential in phage therapy (Fayez et al., 2021; Supplementary Table S9). Furthermore, in the one-step growth curve experiment, KP1 and KP12 showed similar latent periods yet higher burst sizes than the previously reported *Klebsiella* phages (Karumidze et al., 2013; Ciacci et al., 2018; Li et al., 2020). As of now, further studies such as *K. pneumoniae* 77 capsular serotype reference strains (Brisse et al., 2004) for host specificity test, capsular typing with *wzc* and *wzy* sequencing for the tested ESBL (+) *K. pneumoniae* strains (Pan et al., 2015), or whole genome sequence-based locus typing of the bacterial strains by public available tool (Kaptive 2.0; Lam et al., 2022), would be required to further characterize the application scope of these two phages.

The effectiveness of phage applications against pathogens is influenced by the survival and persistence of bacteriophages which in turn is highly dependent on physicochemical factors such as pH and temperature. For instance, the viability of *Salmonella* bacteriophage may be affected during oral administration which exposes them to the low pH of the stomach/gizzard (Gigante and Atterbury, 2019). KP1 and KP12 showed higher heat and pH stability (temperature: up to 60°C, pH: 4–11) as compared to the previously reported *Klebsiella* phages (Karumidze et al., 2013; Ciacci et al., 2018; Li et al., 2020). Combined results of host range and physio-chemical characteristics suggested that both KP1 and KP12 have the potential to be novel candidates for therapeutic agents against the antibiotic-resistant *K. pneumoniae*.

Phages evolve mechanisms that overcome the layers of bacterial cell envelope to first, deliver the genetic material to the host cell cytoplasm and subsequently, lyse the host cell wall of PG to release its newly formed virions at the end of a phage lytic cycle. While different lysis pathways have been described, tailed bacteriophages often adopt a concerted holin-endolysin strategy (Fernandes and Sao-Jose, 2018). Holins are small membrane proteins that form holes in the bacterial membrane and trigger the host cytoplasmic disruption that allows the accumulated endolysins to access their PG substrate. Endolysins, enzymes that cleave cell wall PG, are typically categorized into four groups according to their specific activity against different covalent linkages: (i) glycosylase, (ii) transglycosylase targeting the glycosidic linkages, (iii) amidase, and (iv) endopeptidase targeting the oligopeptide cross-linkages. This specific lytic capacity has rendered phage-encoded endolysins its high value in biotechnology applications (Schmelcher et al., 2012). In Gram-negative bacteria, spanin—the third class of lysis proteins often forms a lysis cassette with holin and endolysin, is responsible for degradation of the final outer membrane barrier (Cahill and Young, 2019). As expected, both KP1 and KP12 harbor host lysis PEGs so that they have lytic activities against *K. pneumoniae*, a typical Gram-negative pathogen.

Phage-encoded endolysin has been a promising antibacterial agent because of its advantages compared to phage itself such as non-proliferation, rapid bacterial cell lysis, low bacterial resistance, clear mode of action, easy-to-engineer, anti-biofilm property, and synergistic activity with different antibacterial agents (Schmelcher et al., 2012; Abdelrahman et al., 2021; Lu et al., 2021). To date, several endolysin-based therapies are in clinical trials to demonstrate its safety and efficacy (Kim et al., 2018; Wang et al., 2021), indicating its prevalence as a major antibiotics alternative to treat multidrug-resistant bacteria. Albeit endolysins represent conserved functions across diverse strains of bacteriophages, as demonstrated here, they can have significant variability at the amino acid level. The variability observed within endolysins was suggested to grant them their host specificity and efficiency (Fernandez-Ruiz et al., 2018). Compared to the endolysin sequences of *Klebsiella* phages, KP1.peg.110 revealed high homology with endolysin of Mineola phage that belongs to the *Jiaodavirus* genus. On the other hand, KP12.peg.319 was closely related to SAR endolysin sequences with a distinct sequence organization. The endolysin infecting Gram-negative bacteria are mostly single-module, globular protein (about 20 kDa) with its full length mainly covered by enzymatically active domains whereas the lytic proteins from a Gram-positive background are separated into two domains for cell wall binding and enzymatically active site (Broendum et al., 2018). Consistent with the previous studies (Nelson et al., 2012; Maciejewska et al., 2017; Plotka et al., 2019), molecular weights of putative endolysins KP1.peg.110 and KP12.peg.319 are 18.06 kDa and 18.7 kDa, respectively.

AlphaFold, a recently advanced neural network-based model, has allowed us to predict 3D protein structure with remarkable accuracy from just the target amino acid sequences (Jumper et al., 2021). In this study, the predicted 3D models for phage putative lysis proteins such as endolysin and anti-/holin were generated. In the case of endolysin, computational structure prediction has proven to be particularly useful as it circumvents the challenge of structural hindrance in crystal formation caused by short flexible linker region between domains when conducting X-ray crystallography (Love et al., 2018). In the AlphaFold models, regions with pLDDT above 90 are expected to be modeled with high accuracy, and regions with pLDDT between 70 and 90 generally have the correct backbone prediction (Tunyasuvunakool et al., 2021). Therefore, endolysin model predictions with the average pLDDT above 90 were considered reliable. Both pLDDT and predicted TM-scores of KP1.peg.110 and KP12.peg.319 were about 90, suggesting that their AlphaFold models were of high accuracy. TM-align analysis of KP1.peg.110 (0.96297 for 102 l) and KP12.peg.319 (0.83277 for 1XJT) showed the highest TM-align scores among the candidates (Broendum et al., 2018). Together with the lysis cassette architecture found in their genomes, AlphaFold predictions suggested that KP1 and KP12 harbor distinct lysis-associated proteins of T4-like bacteriophage and P1/P2-like bacteriophage, respectively. Regrettably, the lytic activity of these putative endolysins on Gram-negative bacteria has not been experimentally verified. Further investigation of their purified or recombinant-producing form *in vitro* and *in vivo* would undoubtedly provide useful insights. Nevertheless, the 3D structure models and their comparative analyses in this study may contribute to their potential in designing novel enzybiotics (enzyme-based antibacterials) targeting ESBL(+) *K. pneumoniae*. Indeed, AlphaFold model prediction has facilitated study of phage structures including adhesion devices of phage (Goulet and Cambillau, 2021).

Taken together, this study characterized two *Klebsiella* phages KP1 and KP12. A combination of the genomic and morphological analysis showed that both bacteriophages are members of *Myoviridae* family and are distantly related as they belong to different genera, *Jiaodavirus* for KP1 and *Mydovirus* for KP12. Their physiological features indicated desired lytic activity, stability, and life cycle for controlling ESBL(+) *K. pneumoniae*. A look into endolysin homologous sequences among *Klebsiella* phages revealed an interesting find of vast diversity and possible analogous convergence, which challenge our current understanding of this enzyme. Deep learning-based modeling approach followed by a residue-to-residue alignment of predicted 3D structures to experimentally verified X-ray crystals has enabled a comprehensive understanding of not only phage endolysin but also other representative phage lytic proteins such as holin, i-/o-spanin, or anti-holin. An integrated approach of physiological evaluations and comparative genomic analyses supported by rapid structural prediction would certainly expedite functional annotation of phages and their high-value phage-encoded enzymes. With the desired stability and lytic activity against a broad range of *K. pneumoniae,* KP1 and KP12 themselves and their endolysins have proven their prospects in phage therapy.

## Data availability statement

The datasets presented in this study can be found at https://github.com/SBML-Kimlab/KP1_KP12.

## Author contributions

YK designed and conducted *in vivo* bacteriophage experiment and wrote the manuscript. S-ML analyzed data (i.e., *in silico* comparative genomic analysis and protein structure prediction analysis) and wrote the manuscript. LN analyzed data (*in silico* comparative genomic analysis) and wrote the manuscript. JK assisted the data analysis and revised the manuscript. SK discussed the results and supervised the project. DK discussed the results, corrected the manuscript, and supervised the project. All authors contributed to the article and approved the submitted version.

## Conflict of interest

YK was employed by Optipharm Inc.

The remaining authors declare that the research was conducted in the absence of any commercial or financial relationships that could be construed as a potential conflict of interest.

## Publisher’s note

All claims expressed in this article are solely those of the authors and do not necessarily represent those of their affiliated organizations, or those of the publisher, the editors and the reviewers. Any product that may be evaluated in this article, or claim that may be made by its manufacturer, is not guaranteed or endorsed by the publisher.

## Footnotes

^1^ https://www.patricbrc.org

^2^ https://github.com/deepmind/alphafold

## Supplementary material

SUPPLEMENTARY FIGURE S1 Plaque morphology and protein pattern analysis of bacteriophages KP1 and KP12. Plaque morphology and protein pattern of KP1 (A,B) and KP12 (C,D) were investigated. To observe plaque morphology, bacteriophage and its host bacteria were mixed, incubated, and photographed after 24 hours for KP1 (A) and KP12 (C). Protein patterns for KP1 (D) and KP12 (D) were analyzed using SDS-PAGE of their purified proteins. Major proteins of indicated size were observed. Lane M: protein marker.

SUPPLEMENTARY FIGURE S2 Phylogenetic analysis of 135 *Klebsiella* phages including KP1 and KP12. Tree was constructed based on the phage amino acid sequences, using BPGA pipeline (USEARCH clustering tool with identity cut-off = 50%, MUSCLE alignment and UPGMA algorithm). KP1 and KP12 were observed to be closely related to phages of *Myoviridae* family.

SUPPLEMENTARY FIGURE S3 Core-Pan plots of *Klebsiella* phages. The plots represent the open pan-genome of 135 *Klebsiella* phages (A), *Ackermannviridae* (B), *Autographiviridae* (C), *Demerecviridae* (D), *Drexlerviridae* (E), *Myoviridae* (F), *Poroviridae* (G), *Schitoviridae* (H) and *Siphoviridae* (I). *n* indicates the number of strains for each family.

SUPPLEMENTARY FIGURE S4 Phylogenetic tree of endolysin amino acid sequences of 70 *Klebsiella* phages. Phylogenetic tree was constructed using putative endolysin sequences of KP1 (KP1.peg.110), KP12 (KP12.peg.319), and 68 amino acid sequences annotated as endolysin in NCBI-deposited *Klebsiella* phages. All labels except KP1 (KP1.peg.110, highlighted in red) and KP12 (KP12.peg.319, highlighted in blue), contain NCBI protein ID (YP_#), its product and its origin phage. Multiple sequence alignment and phylogenetic analysis were conducted with ClustalW alignment with maximum likelihood clustering algorithm with 100 iterations.

SUPPLEMENTARY FIGURE S5 Structural prediction of the remaining putative endolysin sequences of KP1 and KP12. (A) AlphaFold models generated for KP1.peg.142, KP12.peg.198 and KP12.peg.279. Protein surface structure of the first pTM model (model_1) (top left), and its corresponding 2D plot showing the score for amino acid at each position (top right) are color-coded according to its pLDDT. PAE heatmaps of five models and their pTM scores illustrate the predicted error between all pairs of residues. The lower value (darker green) indicates a higher confidence in the prediction.

SUPPLEMENTARY FIGURE S6 Structure prediction of putative tail lysozyme subunit in KP1 (KP1.peg.142) and spanin in KP12 (KP12.peg.278). The structures of KP1.peg.142 (A) and KP12.peg.278 (B) were predicted using AlphaFold. Protein surface structure is color-coded according to the pLDDT scores of its first pTM model (model_1).

SUPPLEMENTARY FIGURE S7 Validation of lytic capacity of putative endolysins in KP1 and KP12. Overexpression and purification of the putative endolysin in KP1 (A) and KP12 (B). The information for each lane is described in the figures. (C) Spot tests of purified endolysins in *K. pneumoniae* lawn. Each row indicates spot test results from biological triplicates of purified endolysin in KP1 (upper row), KP12 (middle row), and blank buffer (lower row), respectively.
