# Supplementary figures and images for: Characterization of Klebsiella pneumoniae bacteriophages, KP1 and KP12, with deep learning-based structure prediction
Source: Front Microbiol. 2023 Jan 24;13:990910. doi: 10.3389/fmicb.2022.990910 (PMC9902359; doi:10.3389/fmicb.2022.990910)

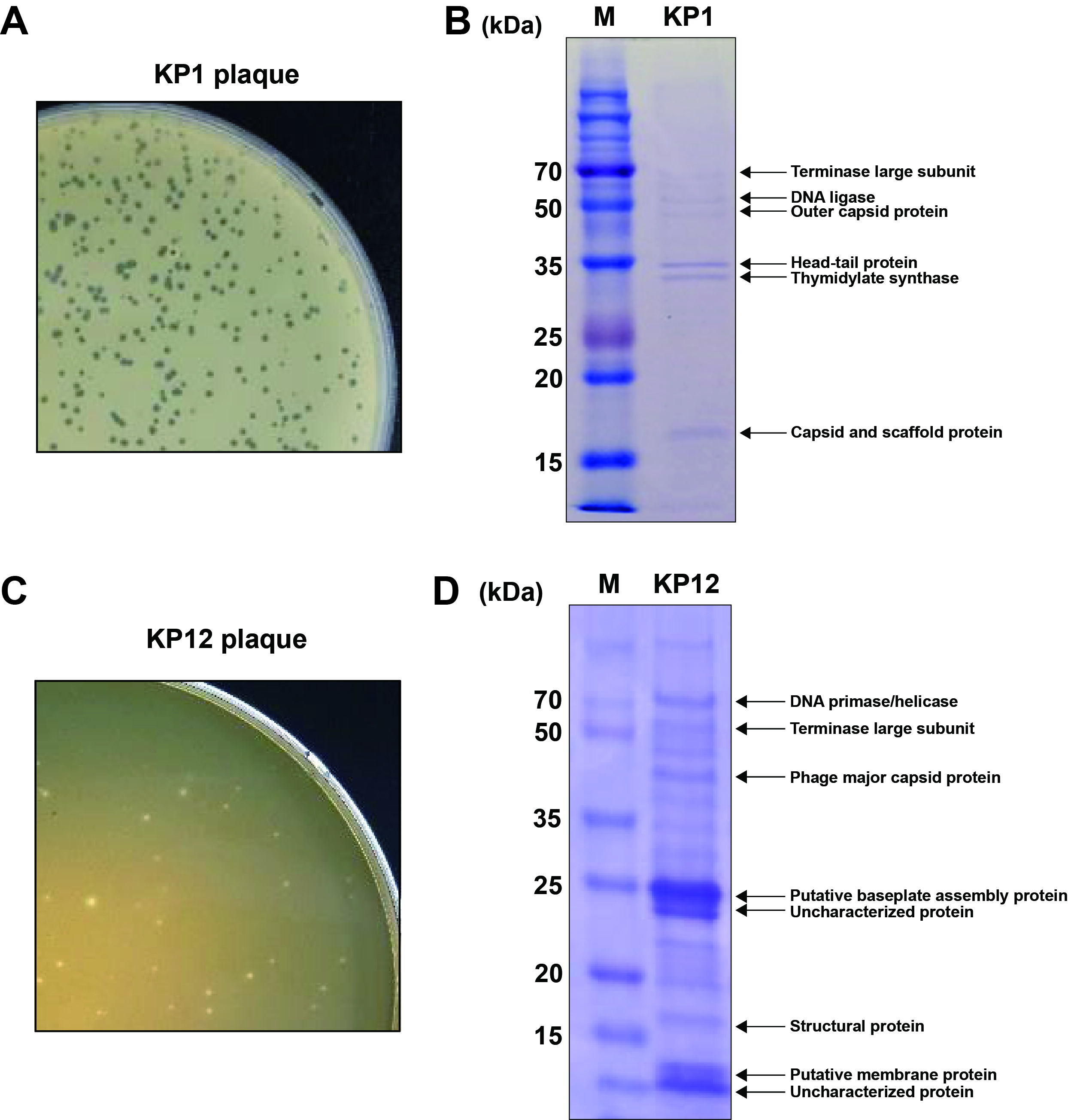

Supplement: SUPPLEMENTARY FIGURE S1 — Plaque morphology and protein pattern analysis of bacteriophages KP1 and KP12. Plaque morphology and protein pattern of KP1 (A,B) and KP12 (C,D) were investigated. To observe plaque morphology, bacteriophage and its host bacteria were mixed, incubated, and photographed after 24 hours for KP1 (A) and KP12 (C). Protein patterns for KP1 (D) and KP12 (D) were analyzed using SDS-PAGE of their purified proteins. Major proteins of indicated size were observed. Lane M: protein marker. [file Image_1.TIF]

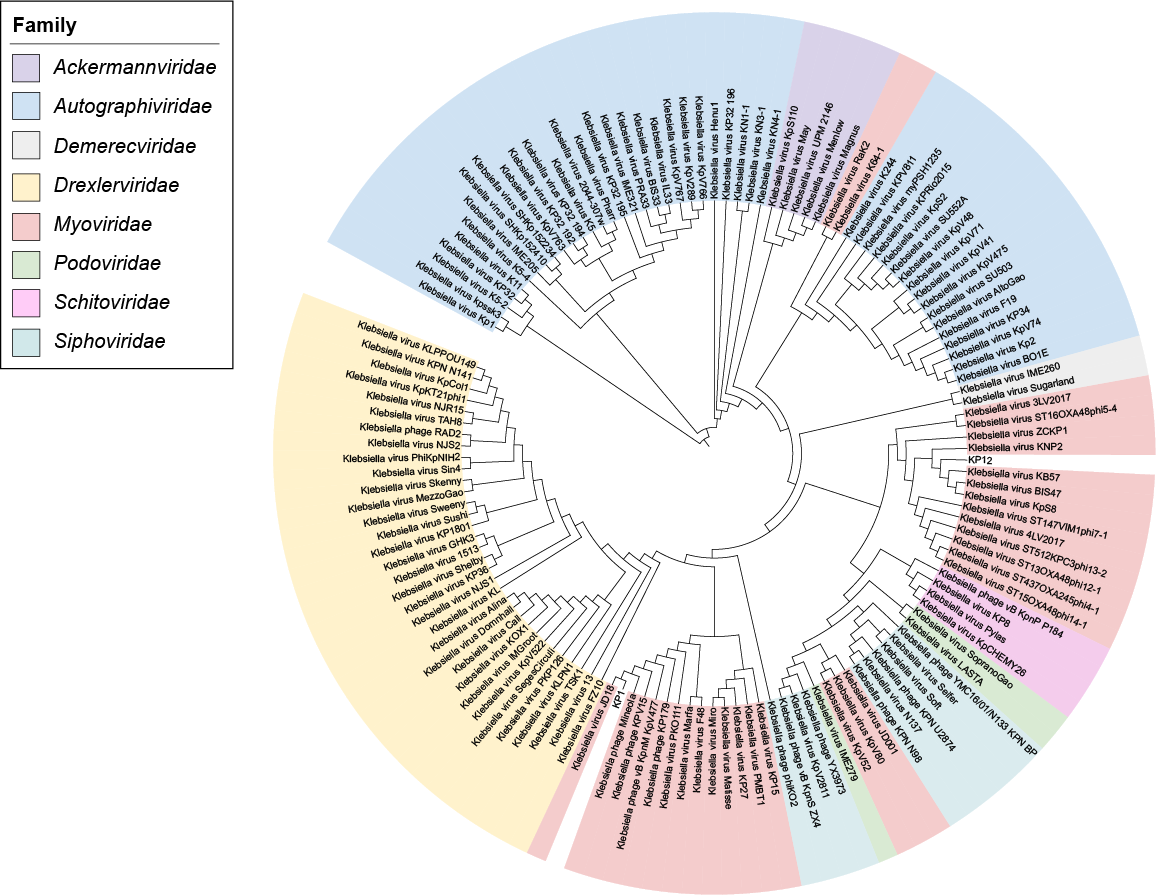

Supplement: SUPPLEMENTARY FIGURE S2 — Phylogenetic analysis of 135 Klebsiella phages including KP1 and KP12. Tree was constructed based on the phage amino acid sequences, using BPGA pipeline (USEARCH clustering tool with identity cut-off = 50%, MUSCLE alignment and UPGMA algorithm). KP1 and KP12 were observed to be closely related to phages of Myoviridae family. [file Image_2.TIF]

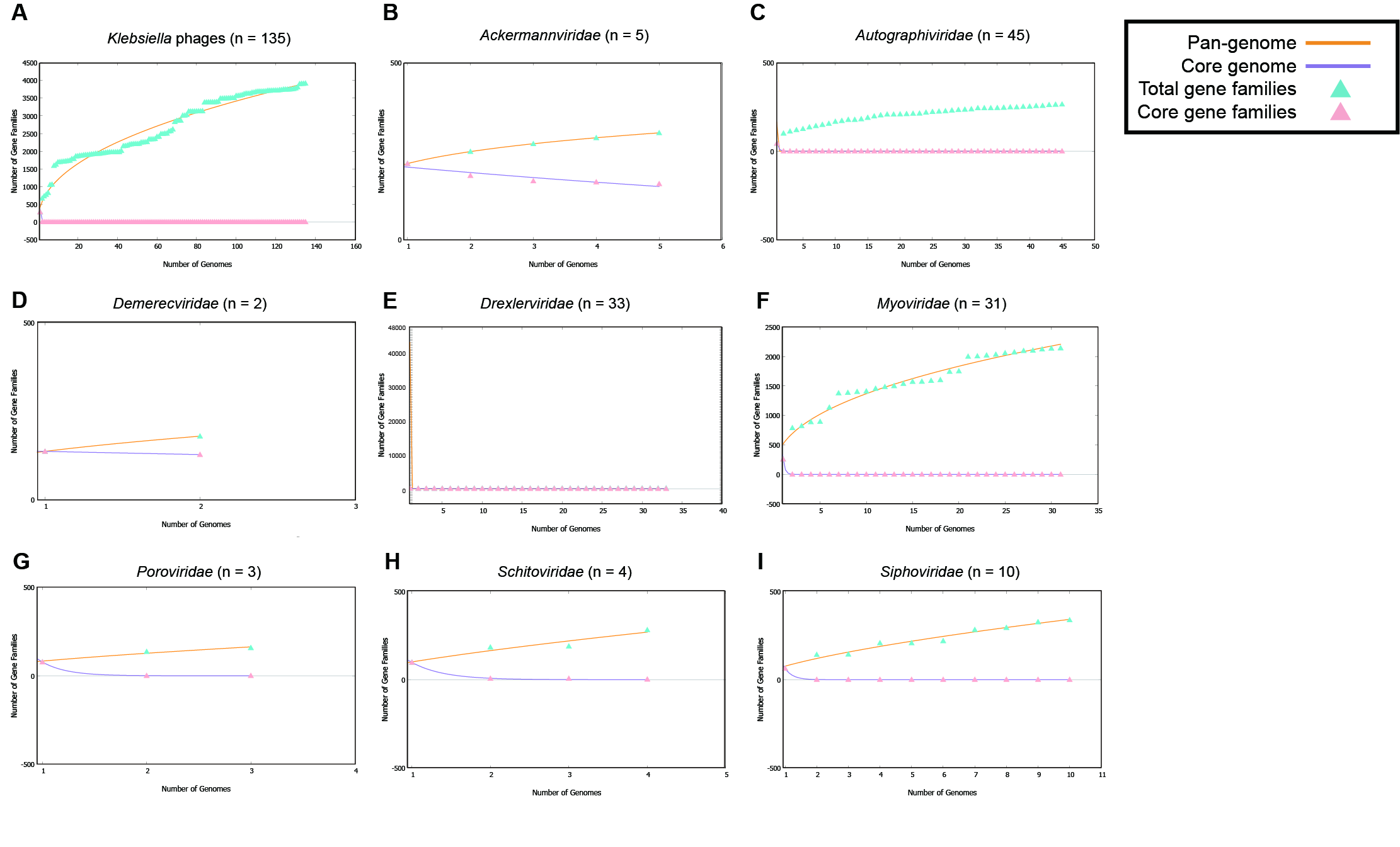

Supplement: SUPPLEMENTARY FIGURE S3 — Core-Pan plots of Klebsiella phages. The plots represent the open pan-genome of 135 Klebsiella phages (A), Ackermannviridae (B), Autographiviridae (C), Demerecviridae (D), Drexlerviridae (E), Myoviridae (F), Poroviridae (G), Schitoviridae (H) and Siphoviridae (I). n indicates the number of strains for each family. [file Image_3.TIF]

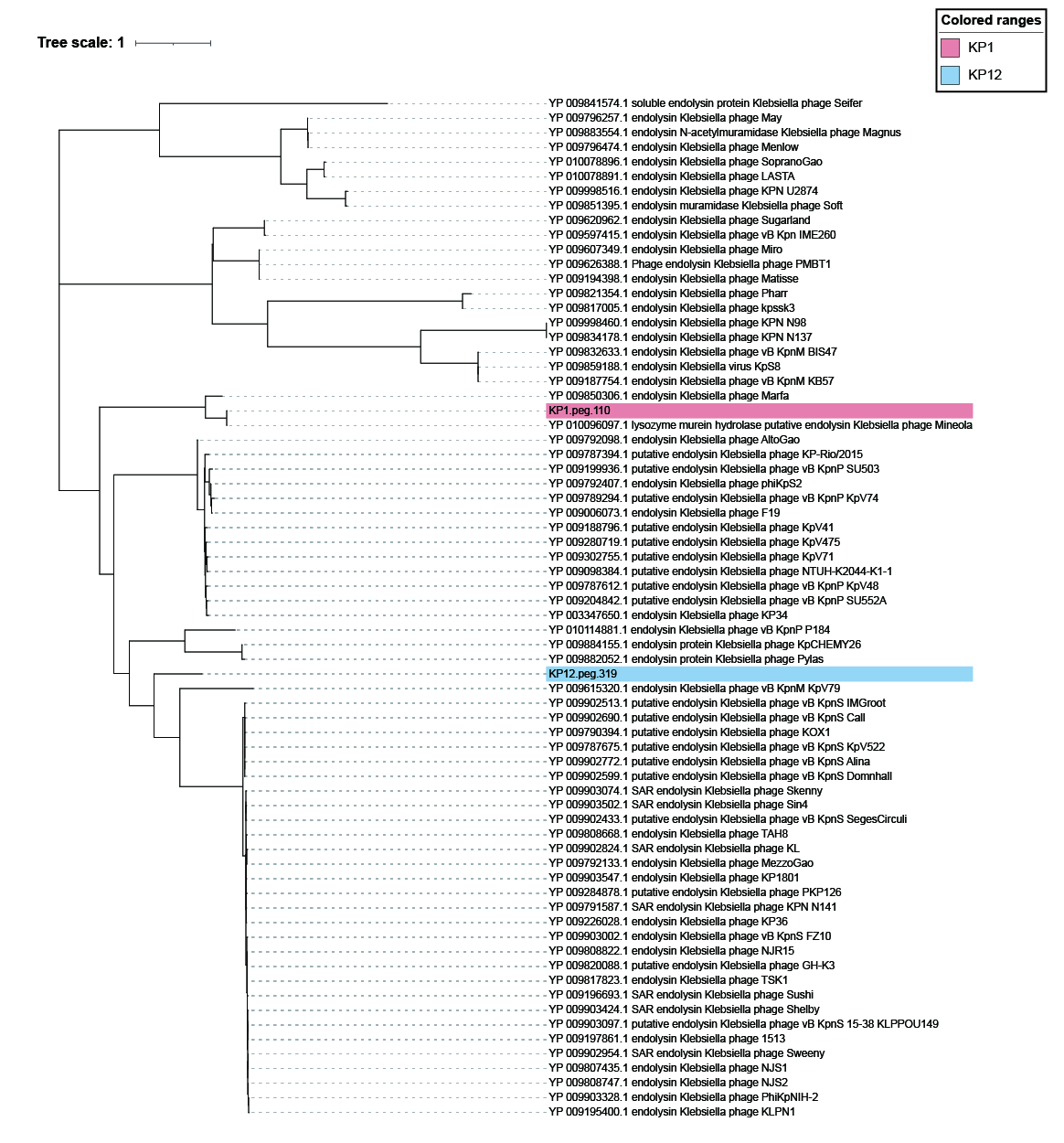

Supplement: SUPPLEMENTARY FIGURE S4 — Phylogenetic tree of endolysin amino acid sequences of 70 Klebsiella phages. Phylogenetic tree was constructed using putative endolysin sequences of KP1 (KP1.peg.110), KP12 (KP12.peg.319), and 68 amino acid sequences annotated as endolysin in NCBI-deposited Klebsiella phages. All labels except KP1 (KP1.peg.110, highlighted in red) and KP12 (KP12.peg.319, highlighted in blue), contain NCBI protein ID (YP_#), its product and its origin phage. Multiple sequence alignment and phylogenetic analysis were conducted with ClustalW alignment with maximum likelihood clustering algorithm with 100 iterations. [file Image_4.TIF]

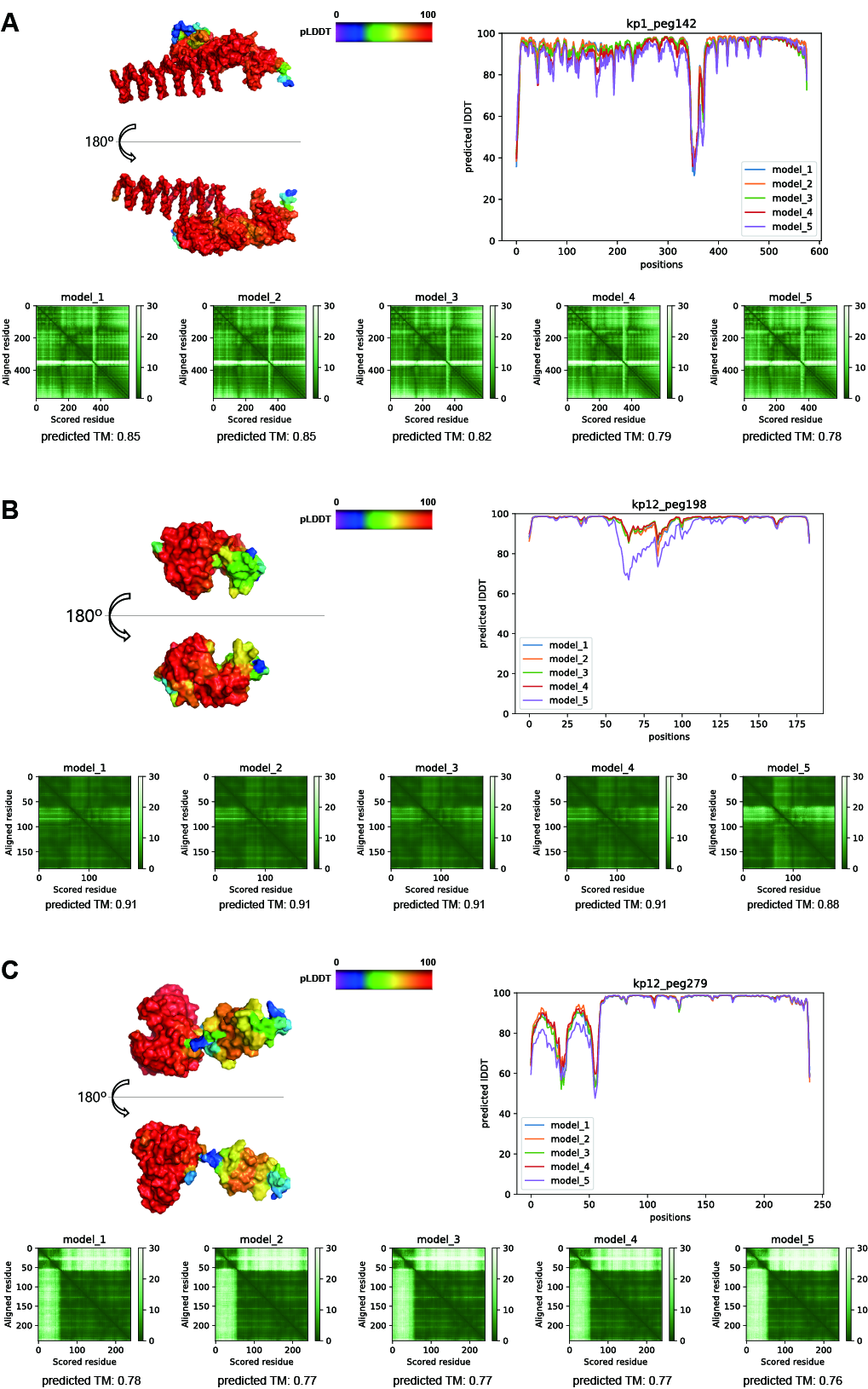

Supplement: SUPPLEMENTARY FIGURE S5 — Structural prediction of the remaining putative endolysin sequences of KP1 and KP12. (A) AlphaFold models generated for KP1.peg.142, KP12.peg.198 and KP12.peg.279. Protein surface structure of the first pTM model (model_1) (top left), and its corresponding 2D plot showing the score for amino acid at each position (top right) are color-coded according to its pLDDT. PAE heatmaps of five models and their pTM scores illustrate the predicted error between all pairs of residues. The lower value (darker green) indicates a higher confidence in the prediction. [file Image_5.TIF]

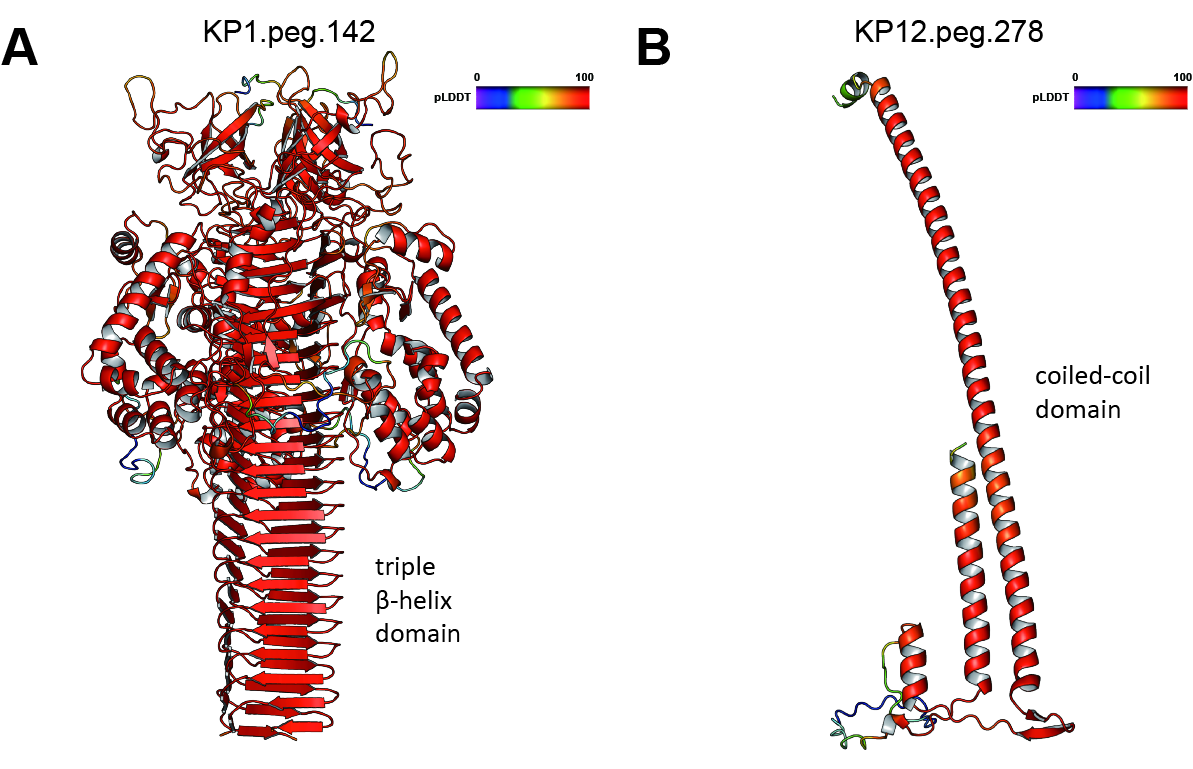

Supplement: SUPPLEMENTARY FIGURE S6 — Structure prediction of putative tail lysozyme subunit in KP1 (KP1.peg.142) and spanin in KP12 (KP12.peg.278). The structures of KP1.peg.142 (A) and KP12.peg.278 (B) were predicted using AlphaFold. Protein surface structure is color-coded according to the pLDDT scores of its first pTM model (model_1). [file Image_6.TIF]

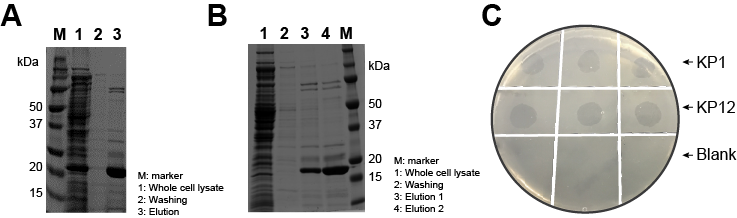

Supplement: SUPPLEMENTARY FIGURE S7 — Validation of lytic capacity of putative endolysins in KP1 and KP12. Overexpression and purification of the putative endolysin in KP1 (A) and KP12 (B). The information for each lane is described in the figures. (C) Spot tests of purified endolysins in K. pneumoniae lawn. Each row indicates spot test results from biological triplicates of purified endolysin in KP1 (upper row), KP12 (middle row), and blank buffer (lower row), respectively. [file Image_7.TIF]
